# Nasal IgA Provides Protection against Human Influenza Challenge in Volunteers with Low Serum Influenza Antibody Titre

**DOI:** 10.3389/fmicb.2017.00900

**Published:** 2017-05-17

**Authors:** Victoria M. W. Gould, James N. Francis, Katie J. Anderson, Bertrand Georges, Alethea V. Cope, John S. Tregoning

**Affiliations:** ^1^Mucosal Infection and Immunity, Section of Virology, Imperial College LondonLondon, United Kingdom; ^2^Altimmune, London BioScience Innovation CentreLondon, United Kingdom

**Keywords:** influenza, IgA, nasal, vaccine, Human Infection Challenge study

## Abstract

In spite of there being a number of vaccines, influenza remains a significant global cause of morbidity and mortality. Understanding more about natural and vaccine induced immune protection against influenza infection would help to develop better vaccines. Virus specific IgG is a known correlate of protection, but other factors may help to reduce viral load or disease severity, for example IgA. In the current study we measured influenza specific responses in a controlled human infection model using influenza A/California/2009 (H1N1) as the challenge agent. Volunteers were pre-selected with low haemagglutination inhibition (HAI) titres in order to ensure a higher proportion of infection; this allowed us to explore the role of other immune correlates. In spite of HAI being uniformly low, there were variable levels of H1N1 specific IgG and IgA prior to infection. There was also a range of disease severity in volunteers allowing us to compare whether differences in systemic and local H1N1 specific IgG and IgA prior to infection affected disease outcome. H1N1 specific IgG level before challenge did not correlate with protection, probably due to the pre-screening for individuals with low HAI. However, the length of time infectious virus was recovered from the nose was reduced in patients with higher pre-existing H1N1 influenza specific nasal IgA or serum IgA. Therefore, IgA contributes to protection against influenza and should be targeted in vaccines.

## Introduction

Better understanding about how individuals are protected against influenza infection will aid vaccine development, by identifying effector mechanisms, and vaccine implementation, by identifying easily measurable correlates of protection (Reber and Katz, 2013). Serum antibodies that bind haemagglutinin and prevent haemagglutination are used as a correlate of protection against influenza: a titre greater than 1:40 in the haemagglutination inhibition (HAI) assay is widely used as a protective value. There are, however, other potentially protective, vaccine-inducible components of the adaptive immune response including mucosal T cells and local IgA (Sridhar et al., 2015).

A range of study types can be used to define correlates of protection. Prospective population surveillance studies can be used as a tool to investigate the correlates of protection (Black et al., 2011), but they are large and expensive and because of the size of the studies, the number of potential correlates measurable may be restricted. An alternative is to use mouse models of influenza infection, which are powerful because all tissues can be sampled, a wide variety of immunological tools are available and more severe infections can be used. But there are differences in the immune response between mouse and man, some of the restriction factors for example MX1 are missing in inbred mouse strains (Pillai et al., 2016) and some strains of virus, for example the current H3N2 strains do not infect mice. An alternative is to use human infection challenge studies: whilst smaller in participant number they allow for an increased strike rate of infection, more intensive sampling and a known start point of infection (Carrat et al., 2008). The HAI titre that is currently used as a correlate of influenza protection was defined in the 1970s in a series of human influenza challenge studies (Hobson et al., 1972).

In the current study, local and systemic antibody correlates of protection were investigated in two human challenge studies. Volunteers were pre-screened for low H1N1 HAI titres and then infected with a virus isolated during the 2009 ‘swine-flu’ pandemic – influenza A/California/2009 (H1N1). Infection was determined by PCR and culture and serum and nasal wash H1N1 specific IgG and IgA measured by ELISA. Serum IgG level prior to infection did not correlate with protection from infection, however, there was a weak but significant correlation between time of virus shedding and serum or local IgA. These results support a protective role for IgA, suggesting that vaccines that can induce it would be protective and that there could be a definable value for IgA that could be used as a correlate of protection.

## Materials and Methods

### Volunteers

The subjects were healthy adults aged 18 to 45 and serologically negative to the challenge virus (serum HAI titres ≤1:10). Detailed inclusion/exclusion criteria are described in clinicaltrials.gov under identifier NCT02014870 and NCT02071329. All subjects gave written informed consent to participate in the trial. The protocol and informed consent were approved by an independent ethics committee, Institutional Review Board, ZNA/OCMW, Antwerp, Belgium. The study was conducted in accordance with EU Directive 2001/20/EC and ICH GCP.

### Clinical Trial

Data from two studies are included here. Clinical challenge volunteers were inoculated with influenza A/California/2009 (H1N1). The first study was a dose escalation study (NCT02014870) and details of this study have been reported elsewhere (Watson et al., 2015): three cohorts were employed: the low cohort were inoculated with 3.5 × 10^4^ TCID_50_, the medium cohort received 3.5 × 10^5^ TCID_50_ and the high cohort received 3.5 × 10^6^ TCID_50_. Subjects entered the quarantine unit 24 h prior to virus challenge and remained for an additional 7 days. The second study is comprised of placebo subjects from a vaccine study (NCT02071329) who were infected with the 3.5 × 10^6^ TCID_50_ of virus. Data from the first study is used to assess sera responses at day 29 after infection, which was not collected in study 2. For the evaluation of protection, data from the high infection cohort and study 2 are pooled for comparable infection dose.

Immediately before challenge, frozen virus was thawed rapidly in a 37°C water bath before dilution in pre-warmed (37°C) phosphate-buffered saline (PBS, Mediatech) to appropriate challenge dose. Subjects were placed in a semi-recumbent position, with the head tipped slightly back and were asked to close their palate. A total of 0.5 ml of diluted virus stock was administered intranasally to each subject (0.25 ml per nostril) using a pipette.

Symptoms were captured twice daily by self-reporting on 16 events associated with influenza illness (headache, nasal stuffiness, runny nose, sore throat, sneezing, hoarseness, earache, facial or eye pain, cough, wheezy chest, breathing difficulty, musculoskeletal ache, nausea/vomiting, feeling/hot/feverish/chills/rigor, fatigue and diarrhea) and classified as absent, mild, moderate or severe. Classifications were defined as follows: mild: the event causes a minor discomfort, does not interfere with daily activity of the subject or does not lead to establishment of a correcting treatment; moderate: the event perturbs the usual activity of the subject and is of a sufficient severity to make the subject uncomfortable; severe: the event prevents any usual routine activity of the patient and causes severe discomfort. In addition, a more objective targeted physical exam was performed twice daily by the study physician which assessed 6 clinical parameters (nasal discharge, otitis, pharyngitis, sinus tenderness, new wheezes, crackles or rhonchi on lung auscultation and percussion) using the same classification system. Vital signs and safety laboratory tests were performed daily. Temperature was measured twice daily. Nasal washes were performed daily by instilling 5 ml of pre-warmed (37°C) sterile phosphate-buffered saline into each nostril and the effluent was collected, diluted 1:1 in virus transport medium and stored at ≤-65°C. Daily nasal-discharge weights were determined by the collection of pre-weighed tissues in pre-weighed plastic bags assigned to each subject during each 24-h period. On the day of discharge from the quarantine unit subjects were required to have negative result from a negative rapid influenza test (Directigen^TM^ EZ Flu A+B, BD).

### Laboratory Analysis

The haemagglutination-inhibition (HAI) assay at Day-1 and 29 (VisMederi srl, Siena, Italy) for all cohorts. Serum was pre-treated with receptor-destroying enzyme (RDE) II (Denka Seiken, Japan) and twofold serially diluted starting from a dilution of 1:10 to 1:2,560 in physiological saline. Virus (A/H1N1/California/04/2009, NIBSC) or (A/Victoria/361/2011) was added to each well at 4 haemagglutination units (HAU)/50 μl for 1 h at room temperature, followed by 0.35% turkey red blood cells for 1 h at room temperature. Reference sheep hyperimmune antisera were provided by NIBSC.

For real-time RT-PCR (rRT-PCR), the WHO protocol “CDC protocol of real-time RT-PCR for swine influenza A (H1N1)” was followed by VisMederi srl using but only the InfA primer designed for the universal detection influenza A viruses. A specimen was considered positive for influenza A if cycle threshold (Ct) values were within Ct values of 40. A virtual quantification tool was applied to the Ct values to allow the conversion of CDC rRT-PCR Ct values to virus RNA copy number, in the absence of a standard curve run in parallel to the samples (Piralla et al., 2013).

### Semi-quantitative Antigen-Specific ELISA

Antibodies specific to influenza were measured using a standardized ELISA (Russell et al., 2016). IgG responses were measured in sera and IgA responses in sera and nasal wash. To detect antigen specific responses, MaxiSorp 96-well plates (Nunc) were coated with 1 μg/ml H1N1 (A/California/09) or H3N2 (A/Texas/50/2012) surface antigens (GSK Vaccines, Sienna, Italy) and incubated overnight at 4°C. Plates were blocked with 1% BSA in PBS. Bound IgG was detected using biotinylated goat anti-human IgG (Sigma) and bound IgA was detected using a biotinylated anti-IgA (AbD Serotec) followed by Poly-HRP40 (Fitzgerald biotech). To quantify the concentration of antigen specific antibody, control wells were coated with a combination of anti-human lambda and kappa light chain specific antibodies (AbDSerotec, Oxford, United Kingdom) and a dilution series of control non-specific human IgG (Sigma) or IgA (Sigma) used as a standard in these wells. TMB with H_2_SO_4_ as stop solution was used to detect the response and optical densities read at 450 nm.

### Statistical Analysis

Correlation of data was performed using Pearson *r*-test. Comparison between 2 values was performed using *t*-tests, comparison of 3 variables was performed using ANOVA and Tukey’s multiple comparison test. All statistical analyses were performed using GraphPad prism 7.

## Results

### Variability in Response to Challenge Infection in Individuals with Low HAI

The aim of the study was to define the antibody correlates of protection against influenza, focusing on IgA. To assess this we measured serum and local antibody responses in volunteers challenged intranasally with 3.5 × 10^6^ TCID_50_ influenza A/California/2009 (H1N1). Clinical challenge volunteers from two studies were used for this study, the first study was a dose escalation and the second study was a vaccine and challenge study; the high dose challenge group from study one was combined with placebo patients from study two (all data is available in **Supplementary Table S1**). Patients were pre-screened for low H1N1 HAI titres (≤10) to increase the likelihood of infection because of the well-established correlation between HAI titre and protection against infection (Hobson et al., 1972).

**FIGURE 1:**
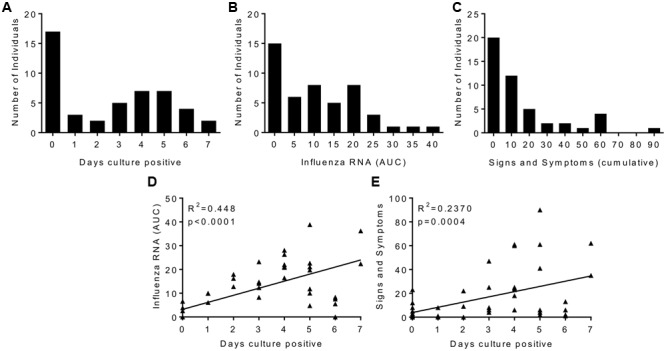
**Human influenza infection in clinical volunteers.** Patients were inoculated intranasally with 3.5 × 10^6^ TCID_50_ influenza A/California/2009 (H1N1). Samples were collected daily and assessed for influenza by culture or rtPCR, and disease signs and symptoms. Cultured virus presented as days virus positive **(A)**, influenza RNA rtPCR data presented as area under the curve plotted over the time course of infection **(B)**, signs and symptoms are presented as cumulative total **(C)**. Correlation plots of days virus positive against detectable Influenza RNA **(D)** or signs and symptoms **(E)**. **(A–C)** Are presented as binned frequencies. Data presented are combined from two studies, *n* = 47 volunteers.

There was a range of outcomes to viral challenge, with 14 out of 47 volunteers having no detectable virus by either PCR or culture. The number of days infected patients had culture positive samples recovered from the nose was normally distributed with the median being 4 days (**Figure 1A**). There was influenza RNA detectable in 33 volunteers (**Figure 1B**). Twenty volunteers showed no signs and symptoms of clinical illness and the median was for a mild response (**Figure 1C**). When plotted as the area under the curve of the RNA detected over the time course of the study, influenza RNA strongly correlated (*r* = 0.4428, *p* < 0.0001) with days virus was detectable (**Figure 1D**). Disease signs and symptoms also correlated with days culture positive (**Figure 1E**). Of the virus negative volunteers, 6 were also asymptomatic, interestingly there were virus positive – asymptomatic patients and virus negative – symptomatic patients. Since all the patients had low HAI titres, the variability in responses points to other host factors contributing to protection against infection.

### Serum IgG is Poorly Predictive of Protection

**FIGURE 2:**
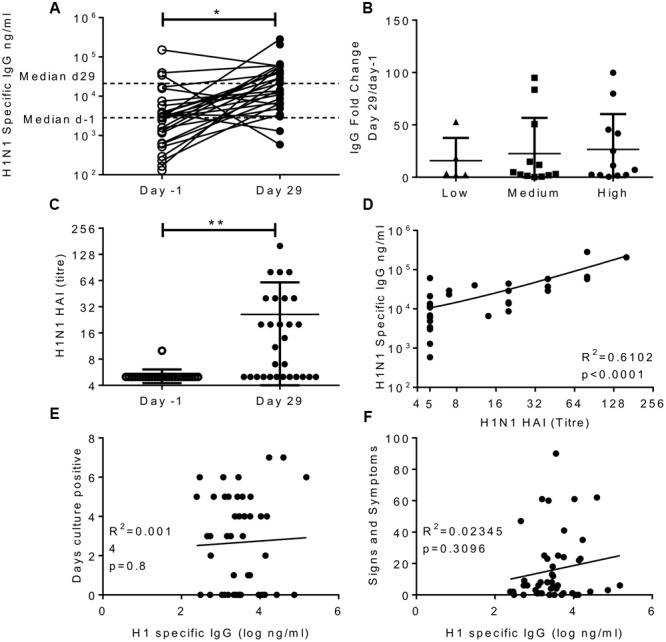
**Infection with influenza increases H1 serum IgG antibody responses, but IgG does not correlate with protection.** Healthy adult human volunteers were infected intranasally with different doses of H1N1 influenza (low dose: 3.5 × 10^4^ TCID_50_, Medium dose: 3.5 × 10^5^ TCID_50_ and high dose: 3.5 × 10^6^ TCID_50_). H1 specific IgG responses were measured by ELISA in serum on day-1 and day 29 of infection and presented as absolute values **(A)**, fold change based on the infecting dose **(B)**. HAI was assessed at day-1 and day 29 **(C)** and compared with IgG titre on day 29 **(D)**. IgG ELISA titre was compared to days culture positive **(E)** and cumulative signs and symptoms **(F)**. **(A–D)** Represent data from a single study *n* = 29 volunteers, **(E,F)** Represent data from two studies combined *n* = 47.

HAI assays measure one facet of antibody immunity, we wished to determine whether H1N1 binding IgG was an independent correlate of protection. Serum was collected 1 day prior to infection and on day 29 after infection in study one only; responses in all three cohorts in this study were compared. There was a significant increase in the H1N1 specific IgG titre in the serum after infection; the median moved from 2,830 to 21,189 ng/ml (**Figure 2A**). The majority of volunteers increased in IgG titre after infection (26 out of 29) and there was no difference in the fold increase in antibody titre between individuals infected with different doses of influenza (**Figure 2B**). Whilst there was detectable IgG in the nose, there was very little H1N1 specific IgG (data not shown). The volunteers all had undetectable HAI prior to challenge, but this significantly increased after challenge (**Figure 2C**), and as seen with IgG challenge dose did not alter the change in HAI. Even though there was a spread of IgG ELISA values, HAI and ELISA IgG did not correlate prior to challenge, presumably as a consequence of pre-screening, but there was a significant correlation between HAI and IgG at day 29 after infection (**Figure 2D**, *R*^2^= 0.61, *p* < 0.0001).

In order to assess possible correlations between IgG and infection, data from the high dose group from the first cohort was combined with subjects from the second study. There was no significant correlation between H1N1 specific IgG and days culture positive (*R*^2^ = 0.0014, *p* = 0.8; **Figure 2E**), viral RNA copies detected by PCR (data not shown) or cumulative signs and symptoms (*R*^2^ = 0.023, *p* = 0.31; **Figure 2F**) infection. This suggests that individuals pre-screened for low HAI also had low serum IgG titres which were also sub-protective, and the two measures do not independently segregate as correlates of protection.

### IgA is Increased by Infection and Weakly Correlates with Protection

**FIGURE 3:**
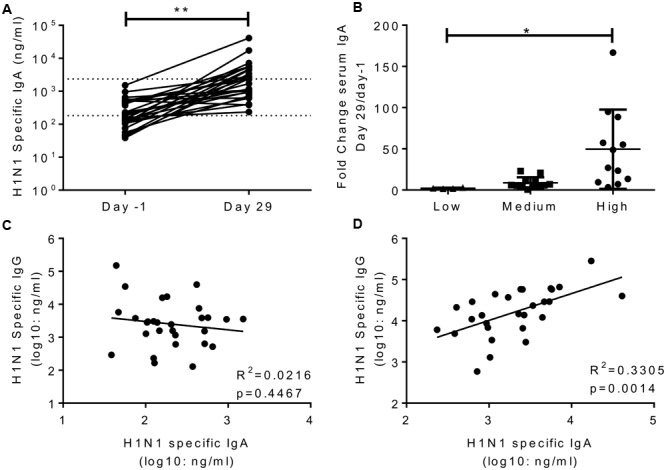
**Infection with influenza increases H1 serum IgA antibody responses.** Healthy adult human volunteers were infected intranasally with different doses of H1N1 influenza. H1 specific IgA responses were measured by ELISA in serum on day-1 and day 29 of infection and presented as absolute values **(A)** and fold change based on the infecting dose **(B)**. Serum IgA and IgG were compared at day-1 **(C)** and day 29 **(D)**. Data from a single study *n* = 29 volunteers.

A number of studies have indicated that IgA is an independent correlate of protection to IgG (Rossen et al., 1970; Clements et al., 1986; Belshe et al., 2000; Ambrose et al., 2012). H1N1 specific IgA was measured in the serum of volunteers in study 1 on day-1 and day 29. H1N1 specific IgA in the serum significantly increased after infection (**Figure 3A**), the average fold change was 24. Interestingly, challenge dose had a significant effect on the increase in IgA titre, with the IgA increasing more in recipients of higher titre virus than those receiving a low dose (**Figure 3B**). Prior to infection, there was no correlation between serum IgA and IgG (**Figure 3C**). However, IgA and IgG did correlate on day 29 after infection (**Figure 3D**). There was no correlation between HAI and IgA (*R*^2^ = 0.03, *p* = 0.384, data not shown).

**FIGURE 4:**
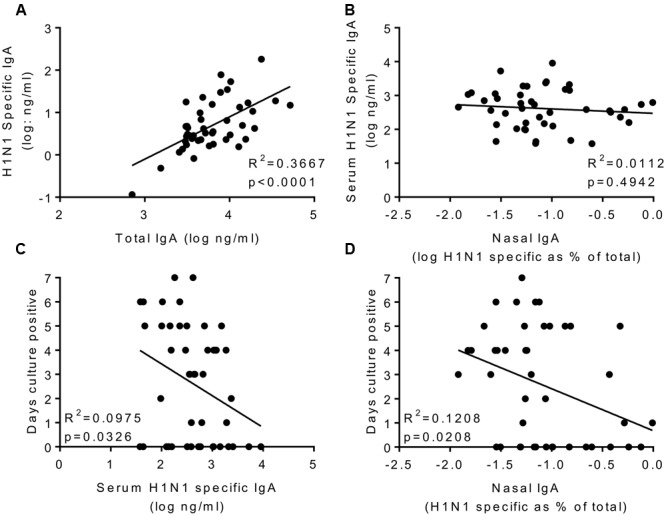
**H1N1 specific IgA in nasal wash and serum correlate weakly with protection.** H1N1 specific IgA and total IgA were measured in the nasal wash samples collected on day-1 prior to infection **(A)**. H1N1 specific IgA responses were compared between nasal wash and serum **(B)**. Days culture positive virus was recoverable compared to serum IgA **(C)** and nasal IgA **(D)**. Data pooled from two studies, *n* = 47 volunteers.

The main aim of the study was to determine whether systemic or local IgA correlated with protection. H1 specific IgA was measured in nasal lavage; to compensate for dilution effects we normalized the values against total IgA recovered (**Figure 4A**). Prior to infection, H1N1 specific IgA in nasal wash and serum did not correlate (**Figure 4B**). Levels of serum and nasal IgA were compared with the same measures of infection tested for IgG. For both serum IgA (*R*^2^ = 0.09293, *p* < 0.05; **Figure 4C**) and nasal IgA (*R*^2^ = 0.08655, *p* < 0.05; **Figure 4D**) there was a weak but significant inverse correlation with the number of days detectable virus was shed, measured by culture. There was a similar, but not significant pattern when comparing IgA with either total RNA detection or symptoms. Therefore, IgA provides a separate correlate of protection to IgG against influenza.

### Protection is Independent of the H3 Specific Response

**FIGURE 5:**
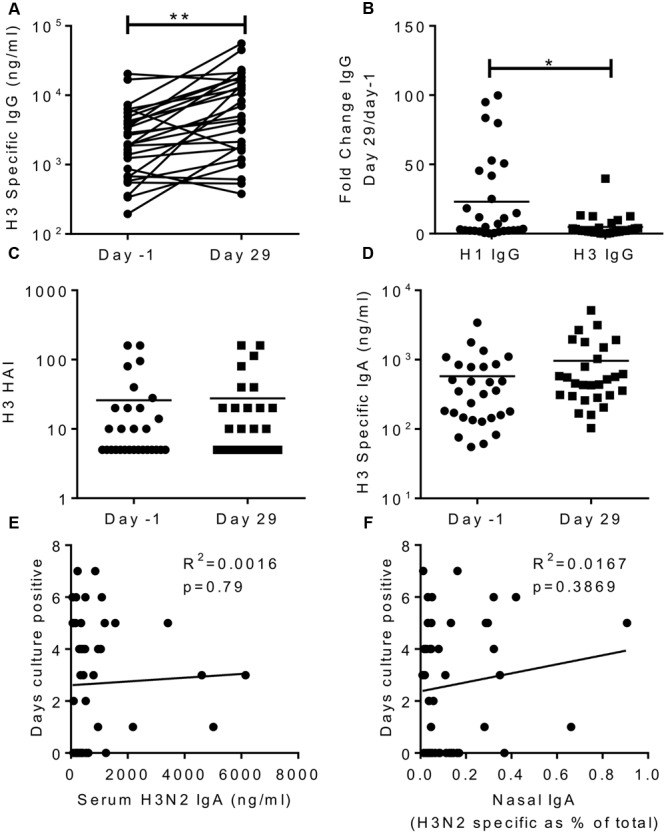
**H3N2 specific antibody does not correlate with protection.** H3N2 specific IgG **(A)**, HAI **(C)** and IgA **(D)** were measured in serum on day-1 and day 29, fold change in IgG response compared between H1N1 and H3N2 IgG **(B)**. Days culture positive virus was recoverable compared to H3N2 specific serum IgA **(E)** and nasal IgA **(F)**. Data pooled from two studies, *n* = 47 volunteers.

To see if the effects we saw were virus serotype specific, we also measured responses against a recently circulating H3N2 virus (A/Texas/50/2012). After infection, there was a significant increase in H3N2 specific IgG in the sera (**Figure 5A**), but this was significantly lower than the H1 fold change (mean of 4 compared to 23 for H1, **Figure 5B**). This slight increase may reflect overlapping antigen similarity between H1 and H3, though there was no correlation between the individuals in terms of their increase in H1 and H3 titres. There was no increase in H3 HAI titre (**Figure 5C**) or H3 IgA in the sera (**Figure 5D**). There was no correlation between H3 IgA in the serum (*R*^2^ = 0.0016, *p* = 0.79; **Figure 5E**) or the nasal lavage (*R*^2^ = 0.0167, *p* = 0.37; **Figure 5F**) and protection against infection.

## Discussion

The aim of the study was to define antibody correlates of protection in a human influenza challenge model. We were able to evaluate the role of IgA in the absence of serum inhibitory antibody because volunteers were pre-screened for low HAI titres. In individuals with low baseline HAI titres, H1N1 strain specific serum IgG measured by ELISA did not correlate with protection against challenge. IgA has previously been proposed as one of the correlates of protection against influenza infection (Rossen et al., 1970; Clements et al., 1986; Belshe et al., 2000; Ambrose et al., 2012). This is supported by murine studies, transfer of nasal IgA from immunized to naïve mice leads to protection against infection (Tamura et al., 1991) and polymeric Ig receptor knockout mice have increased viral load on challenge after intranasal vaccination, with matched virus strains (Asahi et al., 2002). One suggested protective function of influenza specific IgA has been to limit the spread of budding virus from the cell, rather than classical entry inhibiting neutralization (Mazanec et al., 1995), this is supported by studies in guinea pig models demonstrating that IgA prevents influenza transmission (Seibert et al., 2013). In the current study, there was a weak but significant correlation between both nasal lavage and serum IgA and days shedding influenza.

IgA is not the sole protective factor, for example in high dose challenge studies IgA deficient mice had a similar profile on infection as wild type (Mbawuike et al., 1999). Protection also comes from serum IgG transudate into the lungs, which has been observed in both mice (Renegar et al., 2004) and men (Wagner et al., 1987). Serum HAI is a clear correlate of protection against influenza infection (Hobson et al., 1972), though whether the conventional titre of 1:40 is the correct value for all individuals has recently been challenged, especially in more susceptible age groups, for example children (Black et al., 2011). In mouse models the location of the infection determines the functional correlate; IgA is more effective in the upper airway, whilst IgG is more effective in the lower airway (Renegar et al., 2004). The route of antigen exposure affects the type of response, intranasal exposure is more likely to induce IgA, whilst systemic immunization will induce IgG (Clements and Murphy, 1986); in turn, the type of immunity induced has an effect on the correlate of protection –systemic immunization induced protective IgG whilst intranasal immunization induced protective IgA (Clements et al., 1986). In our study we saw that infection boosted influenza specific IgA, as seen in other studies (Wright et al., 1983; Brown et al., 1985), and also IgG. Interestingly, prior to infection IgA and IgG did not correlate, indicating that the different antibody classes can be induced independently. These data suggest IgG and IgA work together to prevent infection.

There are also likely to be factors other than antibody that protect against infection. Of the 47 volunteers challenged in the two studies, 14 had no evidence of infection. These individuals did not have a different distribution of IgA or IgG titres prior to infection to the infected individuals. This suggests that there is not an absolute cut off value of protection, rather that there is a diminishing likelihood of infection as antibody levels increase, this is also seen with HAI (Black et al., 2011). A similar distribution of protective values was seen for IgA in respiratory syncytial virus challenge (Habibi et al., 2015). The data also suggest that there may be other factors that contribute to protection at an individual level. This could include cell intrinsic factors that affect the ability of the virus to replicate in the host cells such as IFITM3 (Everitt et al., 2012; Everitt et al., 2013). It will also reflect the cellular response to infection; T cells will also play a role in protection and clearance of infection (Sridhar et al., 2013), we have previously observed a role for T cell immunity as an additional layer of protection if antibody mediated immunity is circumvented by the virus (Lambert et al., 2016). Most likely all components of the immune response work in concert to prevent infection; cell intrinsic immunity, antibody and T cells.

There are some limitations to the study. There was only a small number of patient samples and in order to define a specific correlate of protection, more individuals would be necessary. Whilst beneficial for dissection of the immune response, the deliberate selection of low HAI individuals will remove the protective role of IgG and conclusions about the relative importance of IgA vs IgG cannot be drawn from this. The nasal wash sampling technique can lead to a degree of variability in recovery of material, we normalized this against the total IgA, but there may be more effective methods of recovering nasal IgA for example nasosorption (Bergin et al., 2016). In some ways the human challenge model is not completely reflective of human infection, for example natural infection probably occurs with a much lower dose of infection (Frise et al., 2016). There is striking variability compared to the mouse model, which are given a similar dose of virus (Mastelic Gavillet et al., 2015; Russell et al., 2015; Lambert et al., 2016), but mice are more homogenous and also infection naïve. Combining the human and animal challenge models with natural infection data is most likely to give us the clearest picture of the response to infection.

Understanding what contributes to protection is important to develop and optimize vaccines against influenza infection and to make informed decisions about the vaccination policy. For example, it is not clear what the best practice is for the use of live attenuated influenza vaccine (LAIV); in 2016, the Advisory Committee on Immunization voted down the use of LAIV for the 2016–2017 season in the US due to a lack of protective efficacy in children (Grohskopf et al., 2016). It had been assumed that LAIV works through IgA not IgG (Barría et al., 2013), though recent studies performed in adults, in which LAIV may have reduced efficacy, indicated no clear correlate of protection (Wright et al., 2016). There is also not a simple value of IgA comparable to HAI >1:40 that could be measured in efficacy studies. Our studies suggest that nasal and serum IgA can contribute to protection against influenza and that a vaccine that can induce IgA will be protective but larger studies are needed to determine a specific protective value.

## Author Contributions

VG analyzed the data. JF, BG, and KA coordinated the clinical study and made material available for analysis. AC analyzed the data and developed assays. JT designed study, collated and analyzed data and wrote paper.

## Conflict of Interest Statement

VG, AC, JT do not have a commercial association with this project, JF, KA, and BG are employed by Altimmune, that ran the human challenge study as part of a vaccine trial.

## Supplementary Material

TABLE S1 **Raw ELISA data**.
